# Extraventricular neurocytoma

**DOI:** 10.4322/acr.2021.348

**Published:** 2021-12-21

**Authors:** Gabriele Gaggero, Luca Valle, Jacopo Ferro, Davide Taietti, Bruno Spina

**Affiliations:** 1 Ospedale Policlinico San Martino, IRCCS, Anatomic Pathology Unit, Genoa, Italy; 2 Università di Genova, Scuola di Scienze Mediche e Farmaceutiche, Department of Integrated Surgical and Diagnostic Sciences, Division of Anatomic Pathology, Genoa, Italy; 3 Azienda Socio Sanitaria Territoriale, Ospedale Maggiore, Anatomic Pathology Unit, Crema, Italy

**Keywords:** Neurocytoma, Brain Neoplasms, Neoplasms by Site

Neurocytoma is a rare central nervous system neoplasm that usually arises in an intraventricular location (so-called Central Neurocytoma), but also exists in an extraventricular location (defined as Extraventricular Neurocytoma: EVN).

Clinically EVN manifests as headache, nausea, vomiting, complex partial seizures or focal neurological deficits. In some cases, atypical signs characterizing an aggressive clinical behavior may be present.

Topographically, EVN most frequently occurs as intrahemispheric (particularly in the frontal lobes), followed by the spinal cord. However, rarer sites have been described.^1^

Radiologically, EVN is a well-circumscribed, slow-growing neoplasm, usually hyperintense on T2-weighted MRI and isointense/hypointense with no contrast enhancement on T1-weighted MRI.^2^

Microscopically, EVN consists of small, round cells that show neuronal differentiation, without marked atypia and/or pleomorphism, without vascular endothelial proliferation and/or necrosis and with low mitotic and proliferation indexes. The 2016 World Health Organization classification of tumors of the central nervous system (WHO2016) assigns to EVN histological grade II.^3^

Immunohistochemically, the main marker to identify the neuronal nature of the neoplasm is Synaptophysin, but immunohistochemical positivity for Synaptophysin alone is not sufficient to conclude a diagnosis of EVN. In fact, there are other central nervous system tumors that may express it, including mainly: pilocytic astrocytoma, disembryoplastic neuroepithelial tumor, ganglioglioma, glioneuronal papillary tumor, some oligodendrogliomas and diffuse leptomeningeal glioneuronal tumor.

For this reason, the immunohistochemistry of Synaptophysin should be supplemented with immunohistochemistry for OLIG2 (expression of this marker is usually incompatible with EVN) and molecular investigations to look for IDH mutations (in EVN, IDH must be non-mutated) and/or to look for co-deletion of chromosomes 1p and 19q (AVN must be 1p/19q non-codeleted).^3^

The pictures above refer to the case of an 86-year-old woman who was found unconscious on the ground at her home for no apparent reason. When she was taken to hospital, a febrile state set in but, before further investigation, she died.

**Figure 1:**
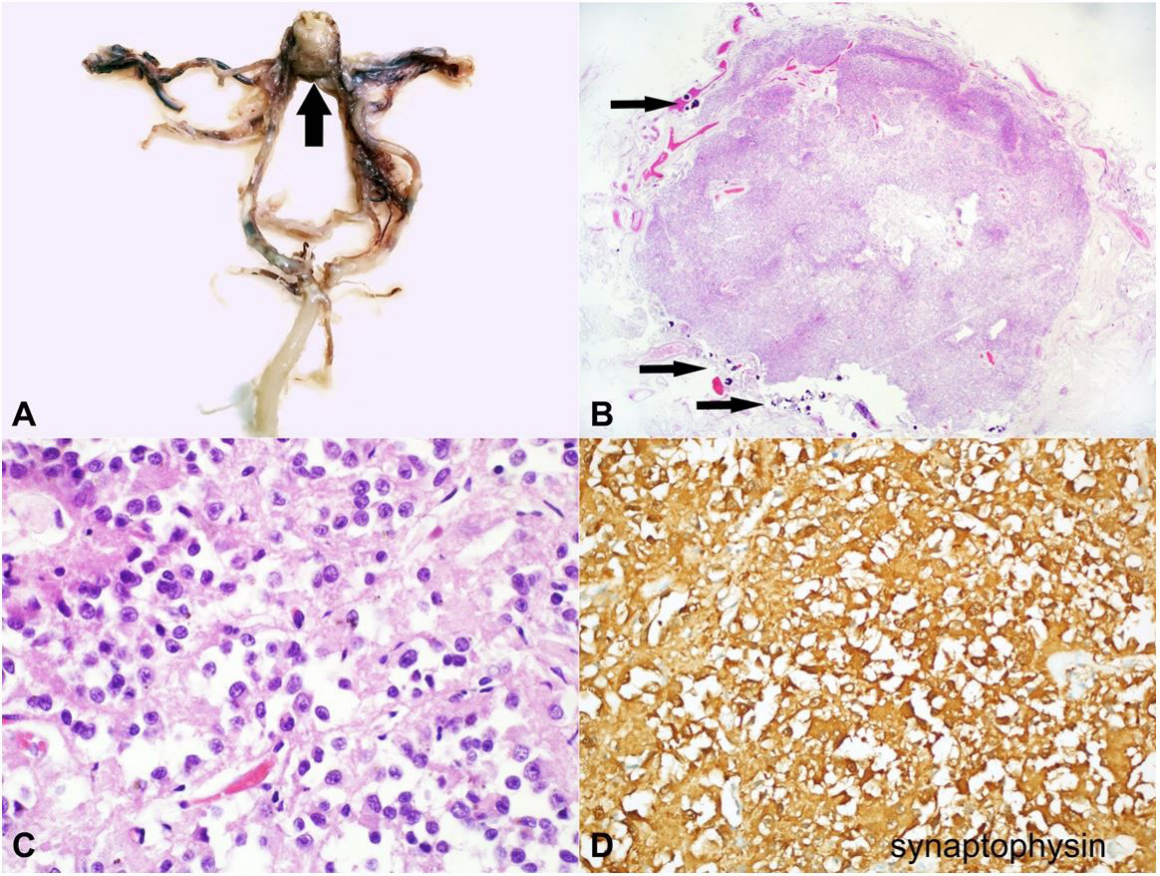
**A** – Macroscopic view of the Willis polygon with a mass at the emergence of the anterior cerebral arteries (arrow); **B** – Photomicrograph of the well- circumscribed nodular lesion with some micro calcifications (arrows) (H&E, 4x); **C** – High power field of the nodular lesion showing round cells, sometimes with evident nucleoli, without significant atypia and without mitosis (H&E; 60x); **D** – Immunohistochemistry, showing positivity for Synaptophysin.

An autopsy was therefore carried out, which revealed acute pneumonia as the main cause of death. However, careful macroscopic examination of the formalin-fixed brain, after isolating the vessels of the polygon of Willis, revealed the presence of a 1.2 cm roundish mass at the bifurcation of the two anterior cerebral arteries (Figure1A).

Histological examination of this lesion showed a nodular proliferation of small, uniform round cells, sometimes with cleared cytoplasm (oligodendroglioma-like appearance), in the absence of mitosis, endothelial proliferation and/or necrosis (Figures 1B, 1C), expressing a neuronal-like immunohistochemical profile: widespread immunohistochemical positivity for S100, Synaptophysin (Figure 1D) and Neuron-specific Enolase (NSE) were detected; the proliferation index, assessed by Ki67, was less than 1%. Immunohistochemical staining was negative for: cytokeratins AE1/AE3; EMA; GFAP; OLIG2; Chromogranin; GH; Prolactin; ACTH; TSH; LH; FSH; TTF1; CD31 and CD34.

Finally, also the immunohistochemical staining for R132-mutant IDH1 protein was negative. The molecular testing (FISH) for 1p/19q deletion status was not performed.

The results were overall consistent with a low-grade neuronal neoplasm of the central nervous system and in particular - although the exceptional site - with an extraventricular neurocytoma.
